# Identifying Leukopenia, Renal Dysfunction and Other Factors Limiting Concomitant Chemoradiotherapy in Locally Advanced Cervical Cancer: A Way to Focus on the Importance of Supportive Care and Potentially Improve Treatment Results?

**DOI:** 10.7759/cureus.90633

**Published:** 2025-08-20

**Authors:** Samuel Vokurka, Lubos Mastny, Radovan Vojtisek, Kateryna Lypka, Jindrich Finek

**Affiliations:** 1 Department of Oncology and Radiotherapeutics, Centre for Palliative and Supportive Medicine, University Hospital and Charles University Faculty of Medicine in Pilsen, Pilsen, CZE; 2 Department of Oncology and Radiotherapeutics, University Hospital and Charles University Faculty of Medicine in Pilsen, Pilsen, CZE

**Keywords:** cervical cancer, chemoradiotherapy, concomitant chemotherapy, leukopenia, renal dysfunction, risk factors, supportive care

## Abstract

Introduction: Concomitant chemoradiotherapy (CHRT) with weekly cisplatin and image-guided adaptive brachytherapy (IGABT) is the standard treatment for unresectable locally advanced cervical cancer. It has been shown that receiving five or more cycles of concomitant chemotherapy (CHT) significantly improves recurrence-free and overall survival. In this study, we analysed the factors contributing to the omission of concomitant chemotherapy within a patient cohort.

Methods: In this single-centre retrospective study, we analysed medical records of 191 patients treated at our facility (Department of Oncology and Radiotherapeutics, University Hospital Pilsen, Czech Republic) from 2012 to 2024 to identify factors preventing optimal CHT administration. Included patients were those with newly diagnosed locally advanced, unresectable cervical cancer, where concomitant platinum-based CHRT with IGABT was indicated.

Results: Of the patients, 15% (29/191) received no concomitant CHT, primarily due to concerns about toxicity, patient frailty/comorbidities, and renal insufficiency, with a statistically significant age difference in those who received CHT versus those who did not: mean 55.03 (±SD11.89) vs. 63.52 (±SD12.14) years (95% CI 3.74-13.23, p=0.0005). Among patients who initiated concomitant CHT but received four or fewer cycles (92/162, 57%), leukopenia was the most common independent limiting factor, followed by decreased renal function. Other factors included nausea, diarrhea, and administrative reasons.

Conclusion: Leukopenia and renal dysfunction were primary barriers to completing optimal concomitant CHT in a significant number of patients. We advocate for increased emphasis on supportive care, including granulocyte colony-stimulating factor (G-CSF) use in leukopenia/neutropenia prevention or treatment, and meticulous management of renal functions. Individualized renal assessment, not just 'cisplatin-unfit' criteria, is crucial. Further research on G-CSFs in CHRT is warranted.

## Introduction

Cervical cancer, a significant and highly preventable malignancy caused by human papillomavirus (HPV) infection, exhibits marked regional incidence disparities due to access to HPV vaccination, cervical screening, and treatment services [1]. Chemoradiotherapy (CHRT) with cisplatin 40 mg/m² weekly and image-guided adaptive brachytherapy (IGABT) is the state of the art in the comprehensive treatment of patients with unresectable locally advanced cervical cancer [2,3]. In a large single-centre analysis [4], excellent treatment outcomes were achieved with magnetic resonance (MR) imaging-based IGABT, with five-year local control rates of 85.5% (95% CI 76%-95%) and five-year overall survival estimates of 63.96% (95% CI 52.94%-74.97%). Patients who received five or more cycles of chemotherapy (CHT) had statistically significantly better three-year recurrence-free survival compared to patients who completed fewer than five cycles (79.07%, 95% CI 60.81-97.34, vs. 58.10%, 95% CI 47.22-68.98; p=0.0185), and these results have been confirmed in a larger cohort of patients with longer follow-up, with statistically significant association of disease-free survival (DFS) and overall survival (OS) [4,5]. The significant difference in these outcomes, depending on whether or not five cycles of CHT were given, necessitates consideration of the potential factors that prevented the administration of CHT. Identifying potentially influenceable factors, such as chemotherapy-induced toxicity, may lead to greater attention and focus on appropriate supportive therapy and care, and potentially to a greater likelihood of completing a higher number of weekly cisplatin administrations.

Supportive care in general holds paramount importance in cancer treatment. Chemotherapy-induced neutropenia/leukopenia is among its several specific challenges, and granulocyte colony-stimulating factors (G-CSFs) play a key role in both prevention and treatment. G-CSFs have a clear impact on overall survival by reducing infectious complications and maintaining the relative dose intensity of chemotherapy, particularly in patients undergoing curative-intent treatment [6-8]. In curative settings, where adherence to treatment intensity is critical for optimal outcomes, as observed in the studies by Vojtíšek et al. [4,5] and also Akyildiz et al. [9], and given the substantial number of patients involved, it is imperative to investigate the root causes that prevent full treatment intensity. Therefore, we conducted a detailed analysis of the factors contributing to the omission of concomitant chemotherapy within the patient cohort of the studies by Vojtíšek et al. [4,5]. We are unaware of any such publications specifically within the cohort of chemoradiotherapy with cisplatin, despite the inherent benefits of such contributions. Furthermore, a novel aspect of our work is that analyses revealing shortcomings in supportive care are not frequently published as single-centre analyses.

## Materials and methods

This was a single-centre retrospective analysis of medical records of patients with newly diagnosed locally advanced unresectable cervical cancer treated with concomitant platinum-based CHRT and IGABT at our institution from 2012 to 2024. The objective was to analyse the reasons for the omission of any concomitant CHT and the omission of more than four cycles of concomitant CHT.

Patients suffering from histologically proven cervical cancer, not suitable for surgery, indicated for radical radiotherapy (Department of Oncology and Radiotherapeutics, University Hospital Pilsen, Czech Republic), and who provided signed informed consent were included, and those not fulfilling the aforementioned criteria were excluded.

All patients provided informed consent for standard CHRT and IGABT treatment. No specific Ethics Committee approval was required for this retrospective observational study. Original patient data were anonymized for this analysis, are stored electronically, and can be released for review as de-identified participant data upon request.

Prior to the initiation of CHRT, all patients underwent a comprehensive physical examination, staging evaluation using computed tomography (CT), positron emission tomography/CT, or positron emission tomography/MR, a complete blood count and biochemistry panel encompassing renal and hepatic function parameters and minerals, and assessment of patient comorbidities. During CHRT, clinical assessments and consultations were conducted at a minimum frequency of once weekly, including monitoring of haematological and biochemical parameters. In patients experiencing complications, these assessments were performed with an increased frequency, with assessments done on a daily basis in some cases.

All patients were treated with pelvic external beam radiotherapy (EBRT). Patients received EBRT that included the paraaortic lymph node regions if lymph node involvement in either the paraaortic or pelvic nodal chains had been confirmed. The prescribed dose of EBRT was 45 Gy. In the presence of lymphadenopathy, the intensity modulated radiotherapy (IMRT) and volumetric modulated arc therapy simultaneous integrated boost (VMAT-SIB) technique were used with two dose levels of 45 Gy/55 Gy in 25 fractions. Lymphadenopathy was not confirmed by biopsy in any patient. The IMRT and the VMAT-SIB plans were normalized to cover ≥99% of the planning target volume (PTV) with 90% of the prescription dose and ≥95% of the PTV with 100% of the prescription dose, and were optimized in the Monaco® treatment system (Electa, Stockholm, Sweden). All sets of plans were created using the same 10 MV photon beams from an Elekta Synergy® (Elekta, Veenendaal, Netherlands) linear accelerator equipped with an 80-leaf multileaf collimator. Patients were treated using either IMRT plans with nine coplanar fields using equally spaced gantry angles, or VMAT plans, which were performed using two coplanar arcs. EBRT was combined with concomitantly administered CHT, cisplatin 40 mg/m^2^ weekly. However, some patients did not receive all possible (maximum six) cycles of CHT. Immediately after the end of EBRT, brachytherapy was administered with a total of four applications planned. In all patients, we administered 3-Tesla MR imaging-based IGABT with the help of the uterovaginal applicators Vienna Ring CT/MR, Interstitial Ring CT/MR or Venezia applicator (Elekta); the treatment planning systems Oncentra Masterplan® and Oncentra Brachy® (Elekta); and high-dose-rate (HDR) after-loading machines MicroSelectron® and Flexitron® (Elekta) with an iridium (^192^Ir) source. The prescribed dose was 7 Gy on the 100% isodose.

Basic statistical univariate analyses (two-tailed unpaired t-test) were performed using GraphPad Prism (GraphPad Software, Inc., San Diego, USA) to test the statistical significance of the difference in the mean age of patients who received versus those who did not receive concomitant CHT. p-values <0.05 were considered statistically significant.

## Results

A total of 191 patients, with a median age of 57 (range 27-87) years, were indicated for concomitant CHRT since 2012. The patients were diagnosed with cervical cancer. Specifically, squamous cell carcinoma accounted for 95.2% of cases, while adenocarcinoma made up 4.8% cases. The tumor grades were distributed as follows: Grade 1 (14.2%), Grade 2 (56%), Grade 3 (18.8%), and Grade X (11%). The FIGO (International Federation of Gynecology and Obstetrics) stages observed were IB1 (2.1%), IIB (27.8%), IIIB (4.1%), IIIC1 (43.4%), IIIC2 (18.9%), IVA (1.6%), and IVB (2.1%). Lymph node involvement was present in 69.1% of cases (N1: 46.1%, N2: 23%), while 30.9% had no nodal involvement (N0).

Of these, 162 (85%) received at least one cycle of concomitant CHT, while 29 (15%) did not receive any CHT; for detailed reasons for CHT omission, see Table 1. A statistically significant difference was observed in the mean age of patients who received versus those who did not receive concomitant CHT: 55.03 (range 27-83, ±SD11.89) years versus 63.52 (range 27-87, ±SD12.14) years (95% CI 3.74-13.23, p=0.0005).

**Table 1:** Detailed reasons for concomitant CHT omission in 29 (29/191, 15%) patients out of those indicated for concomitant chemoradiotherapy CHT: chemotherapy; CKD-EPI: Chronic Kidney Disease Epidemiology Collaboration formula; GFR: glomerular filtration rate; LVEF: left ventricular ejection fraction; MDRD: Modification of Diet in Renal Disease formula; n.a.: not applicable

Reason for CHT omission (in general)	No. of affected patients (%)	Details and comments
Poor general condition, frailty or significant comorbidities (poor performance status) without renal dysfunction	n=13 (13/191, 6.8%)	Ethyl encephalopathy; schizoaffective disorder of mixed type; myocardial infarction recently; ulcerative colitis; cardiomyopathy LVEF 46%; immunosuppression after allogeneic bone marrow transplantation; disorientation and bradypsychomotorism; decompensation of diabetes mellitus; granulomatous pulmonary process; body mass index 45; hepatopathy; dependence on oxygen therapy; cerebral palsy
Renal dysfunction in combination with another factor. Renal function methods, median (mL/s): GFR-cystatin CKD-EPI 0.72 (0.23-0.94); GFR-creatinine CKD-EPI 0.81 (0.14-1.61); GFR-cystatin-creatinine CKD-EPI 0.77 (0.17-1.5); GFR-MDRD 0.74 (0.14-1.5)	n=6 (6/191, 3.1%)	Associated additional co-factors: urinary tract infection, nephrostomy, poor general condition, anaemia from bleeding, leukopenia
Other reasons (independent sole factors)	n=6 (6/191, 3.1%)	Renal insufficiency (GFR 0.83 mL/s); presence of nephrostomy; presence of urinary tract stent and urinary tract infection; neurovegetative asthenia and psychic subcompensation; nausea, vomiting, and diarrhea; gynaecological bleeding
Patient refusal of chemotherapy	n=4 (4/191, 2%)	n.a.

In the group of patients who received concomitant CHT (n=162), 92 (57%) received one to four cycles, and 70 (43%) received five to six cycles. For detailed reasons for the reduced number of CHT cycles (one to four cycles administered), see Table 2. No statistically significant difference was found in the mean age of patients who received one to four cycles versus five to six cycles of CHT: 53.51 (range 29-81, ±SD11.67) years versus 56.18 (range 27-83, ±SD11.98) years; the mean difference was 2.67 years (95% CI -6.38 to 1.04, p=0.1572).

**Table 2:** Detailed reasons for administering a reduced number of cycles (one to four) of concomitant CHT in 92 (57%) of the 162 patients who received CHT CHT: chemotherapy; CKD-EPI: Chronic Kidney Disease Epidemiology Collaboration formula; GFR: glomerular filtration rate; MDRD: Modification of Diet in Renal Disease formula; AST: aspartate transaminase; ALT: alanine transaminase

Reason (factor) for CHT reduction	No. of affected patients and incidence as a sole factor	Comments and details
Leukopenia, median: 2.9 (1.0-3.8) x10^9^/L	n=43 (43/162, 26.5%), n=25	Incidence as a co-factor with other reasons: n=18
Renal insufficiency: renal function methods, median (mL/s): GFR-cystatin CKD-EPI 0.73 (0.4-1.01); GFR-creatinine CKD-EPI 0.82 (0.17-1.35); GFR-cystatin-creatinine CKD-EPI 0.79 (0.25-0.99); GFR-MDRD 0.78 (0.16-1.19)	n=20 (20/162, 12.3%), n=9	Incidence as a co-factor with other reasons: n=11. No significant statistical difference among renal function methods and values (p=0.31-0.89)
Nausea, vomiting	n=15 (15/162, 9.2%), n=5	Incidence as a co-factor with other reasons: n=10
Organizational, logistical and administrative reasons	n=11 (11/162, 6.7%), n=11	Incidence as a co-factor with other reasons: n=0
Thrombocytopenia, median: 75.5 (37-97) x10^9^/L	n=10 (10/162, 6.1%), n=2	Incidence as a co-factor with other reasons: n=8
Diarrhea	n=10 (10/162, 6.1%), n=6	Incidence as a co-factor with other reasons: n=4
Infection: urinary tract infection (n=6), *Clostridium difficile* colitis (n=1), herpetic infection (n=1), wound (n=1)	n=9 (9/162, 5.5 %), n=3	Incidence as a co-factor with other reasons: n=6
Anaemia, haemoglobin median: 93 (70-98) g/L	n=4 (4/162, 2.4%), n=0	Incidence as a co-factor with other reasons: n=4
Other reasons (independent sole factors)	n=7 (7/162, 4.3%), n=7	Procedures and examinations (n=3; breast biopsy, 1; hybrid imaging methods, 2); cisplatin allergy (n=1); refusal of blood and infusions (n=1); neurovegetative asthenia and collapses (n=1); hepatic dysfunction with mild ALT/AST elevation (n=1)
Other reasons (co-factors with other reasons)	n=7 (7/162, 4.3%), n=0	Mineral (hypomagnesemia, hypocalcaemia, hyponatremia) imbalance (n=2); hepatic dysfunction with mild bilirubin, AST/ALT elevation (n=2); neurovegetative asthenia (n=2); gynaecological bleeding with tamponade (n=1)

Among patients who received at least one cycle of concomitant CHT, 155 (95.6%) received cisplatin as the sole chemotherapy agent, and 1 (0.6%) patient received carboplatin due to decreased renal function. A switch from cisplatin to fluorouracil was observed in 1 (1/162) patient, and a switch from cisplatin to carboplatin occurred in 5 (5/162) patients, both due to decreased renal function and urinary infection. In 2 (2/155) patients receiving cisplatin alone, a single dose reduction was performed due to leukopenia and anaemia.

## Discussion

According to the World Health Organization and International Agency for Research on Cancer, cervical cancer is the fourth most common cancer in women worldwide and the eighth most common cancer overall. In 2022, it was estimated that there were 662,000 new cases and 349,000 deaths, 85% of which occurred in developing countries where effective screening programmes and access to vaccinations are lacking. In these countries, cervical cancer is also the leading cause of cancer deaths in women, and the increasing trend in these epidemiological indicators is expected to continue; this makes it a major global health and social problem [1,10]. The Czech Republic, however, is not following global trends in this respect; over the last 25 years, there has been a significant reduction in incidence and a slight decrease in mortality. In 1993, the incidence rate was 22.85 per 100,000 (1214 new cases), while the mortality rate was 8.75 per 100,000 (465 deaths). In 2022, the incidence rate was 13.06 per 100,000 (716 new cases), with a mortality rate of 5 per 100,000 (274 deaths), and cervical cancer was the tenth most common cancer in women [11].

Concomitant CHRT with cisplatin 40 mg/m² weekly and image-guided adaptive brachytherapy is currently the preferred method, yielding very favourable treatment outcomes in patients with unresectable locally advanced cervical cancer [2,3], with five-year local control rates of 85.5% (95% CI 76%-95%) and five-year overall survival estimates of 63.96% (95% CI 52.94%-74.97%). The treatment effect, however, correlates with the number of concurrent chemoradiotherapy cycles, as was observed in a large prospective single-center study by Vojtíšek et al. [4]: patients who received five or more cycles of CHT had statistically significantly better three-year recurrence-free survival compared to patients who completed less than five cycles (79.07%, 95% CI 60.81-97.34, vs. 58.10%, 95% CI 47.22-68.98; p=0.0185). Recently, these results have been confirmed in a larger cohort of patients with longer follow-up, with statistically significant association of DFS and OS with the number of chemotherapy cycles less than five cycles versus five or more cycles (p=0.020 and p=0.006, respectively) with four-year DFS 55.0% (42.2-67.9) versus 76.5% (59.2-93.8) and four-year OS 65.6% (52.9-78.3) vs. 87.2% (70.4-100) [5]. Conclusions regarding the significance of cisplatin chemotherapy dosing in CHRT have also been recently supported by retrospective analysis including 654 patients with stage IB3-IVA disease treated with definitive chemoradiotherapy (external beam pelvic with or without para-aortic radiotherapy and brachytherapy) with concomitant chemotherapy in the form of weekly or once-every-three-weeks cisplatin. A cumulative cisplatin dose of >200 mg, particularly in patients with lymph node metastases, significantly improved overall survival [9]. Respecting any potential risks of chemotherapy, efforts should therefore be made to ensure the highest feasible dose intensity of treatment, maximizing the completion of concurrent chemotherapy. To understand and evaluate major obstacles in delivering optimal concurrent chemotherapy is the prerequisite for focusing our attention and effort on solving them. To address these, we conducted a detailed analysis of a cohort of 191 patients with unresectable locally advanced cervical cancer, who were indicated for concomitant CHRT at our facility (from 2012 to 2024); a vast majority of these patients were included in the CHRT cohort previously treated by Vojtíšek et al. and included in the respective studies [4,5].

The objective of our study was to identify and underscore factors significantly reducing the attainable intensity of concomitant chemotherapy, thereby drawing attention to the imperative role of supportive care. Notably, the predominant issues identified were leukopenia and compromised renal function, both of which are amenable to supportive interventions. This work did not primarily focus on evaluating supportive care interventions. Instead, it aimed to identify critical areas limiting adherence to treatment intensity that are crucial for optimal outcomes in curative chemoradiotherapy treatment. We also sought to discuss and suggest the implementation of corrective measures for this patient cohort and treatment approach. As mentioned in the Introduction section, a novel aspect of our work is that analyses revealing shortcomings in supportive care are not frequently published as single-center analyses, and we are unaware of any such publications specifically within the cohort of chemoradiotherapy with cisplatin.

In our cohort, a total of 191 patients were indicated for CHRT within an intent-to-treat framework, but concomitant CHT was not initiated in 29 (29/191, 15%) of them. The main factors for CHT omission here were concerns about toxicity and manageability in the population of predominantly elderly and frail patients with comorbidities (poor performance status in 13/191, 6.8%), renal insufficiency in combination with some other health factor, and very rarely patient refusal of CHT (4/191, 2%). Upon reviewing Table 1, where these factors are listed in detail, one could individually discuss whether an attempt at CHT should have been made for some patients. However, we believe that solely based on the mentioned factors, without the ability to assess the patient's overall condition and their perspective on the risk-benefit ratio of administering or withholding CHT, it is difficult to determine the justification for not initiating concomitant CHT here.

When evaluating renal function and the risk of cisplatin nephrotoxicity, the concept of patient status as 'cisplatin-unfit' or 'cisplatin-ineligible' can be considered, which is currently applied in the decision-making process for patients with metastatic bladder cancer, as reported by Galsky et al. [12]. This standardization defines patients with an Eastern Cooperative Oncology Group (ECOG) performance status of 2 and/or creatinine clearance <60 mL/min and/or Common Terminology Criteria for Adverse Events (CTCAE) grade ≥2 hearing loss, and/or CTCAE grade ≥2 neuropathy as unfit or ineligible. However, this proposal arose from substantial variability in definitions across numerous studies and among clinical oncologists. There was also variability in the optimal method for assessing renal function, with calculated creatinine clearance being most frequently mentioned (47.7%). There was also variability in what creatinine clearance threshold should be used to define 'unfit' patients, with <60 mL/min mentioned by 41.5%, <55 mL/min by 6.2%, <50 mL/min by 33.8% and <45 mL/min by 18.5% of the panelists [12]. Additionally, it is crucial to discuss whether standard criteria applicable to metastatic urothelial carcinoma can be extrapolated to patients with a prognostically more favourable situation, where the administration or withholding of cisplatin has a significant impact on treatment outcomes, such as in the case of CHRT and our patient population.

However, a more significant discussion arises from the evaluation of the cohort of patients who started concomitant CHT but received a maximum of 'only' four cycles of treatment. Within this group of 92 patients, in 57, the main reason for not administering chemotherapy was a single significant factor, while in others, it was more of a combination of several factors.

Leukopenia was identified as the most common independent (n=25) and associated factor (n=18), and in this context, the question arises as to what extent the appropriate use of G-CSFs supports the maintenance of acceptable leukocyte counts or ensure prophylaxis of all neutropenia, febrile neutropenia, subsequent infections and maintaining the relative dose intensity of CHT -- factors that have a clear impact on overall survival, particularly in patients undergoing curative-intent treatment [6-8]. In contrast to the situation in patients treated with chemotherapy at higher risk of febrile neutropenia and severe neutropenia, where recommendations for the prophylactic use of G-CSFs are relatively well-defined [13-16], the situation in patients treated with radiotherapy or CHRT is less precise. However, recent reviews and meta-analyses have provided increasing evidence demonstrating the safety and efficacy of G-CSF use in patients undergoing CHRT [17,18]. Specifically, for example, the prophylactic use of G-CSF (filgrastim, lenograstim, pegfilgrastim) during CHRT for patients with small cell lung cancer promoted a significant reduction in the incidence of neutropenia without any increase in hematologic or radiotherapy-related toxicity [18]. Considering the 'weekly' platinum derivative CHT protocol in locally advanced cervical cancer patients, the use of short-term filgrastim with regular monitoring of complete blood count, particularly leukocyte count trends, could be a suitable approach. In our cohort, we identified 6 out of 43 patients with leukopenia, in whom G-CSFs were used (filgrastim in five and lipegfilgrastim in one), and observed no atypical safety risks in either the short or long term. It can be anticipated that the results of further analyses focused on the use of G-CSFs during CHRT may further contribute to the increased utilization of growth factors, thereby reducing the risks of leukopenia and neutropenia, and consequently, helping to maintain the dose intensity of concurrent CHT.

Another significant factor, either alone or in combination, is a decrease in renal function, a typical toxicity of cisplatin. Here again, discussions can be held on several levels. The reliability and limitations of renal function calculation methodologies within standard formulas must be considered, together with polemics about the variability in the optimal methods for assessing renal function, variability in what creatinine clearance threshold should be used, and a combination of other factors to define cisplatin-unfit or ineligible patients, as discussed above. Furthermore, questions arise regarding the possibility of preventing and treating renal insufficiency (hydration, omission of nephrotoxic drugs) and adjusting the dosage of cisplatin or using carboplatin instead, which was only occasionally used in a few patients: 95.6% (155) received solely cisplatin, in 6, a switch from cisplatin to carboplatin or fluorouracile occurred, and in only 2 patients cisplatin single dose reduction was performed, due to leukopenia and anaemia. Given that the standard of renal function testing changed at our institution during the observation period, we cannot precisely correlate the individual methods. However, we did not observe any major statistically significant differences when comparing them in general. In a few patients, cystatin C indicated significantly lower renal function compared to other methods used concurrently. Therefore, we consider creatinine CKD-EPI (Chronic Kidney Disease Epidemiology Collaboration) a generally accepted standard formula. In the context of the impact of not administering CHT, in cases of uncertainty about the severity of renal insufficiency, a comprehensive assessment of the situation is necessary. This includes verifying the availability of hydration support, excluding nephrotoxic drugs, and possibly consulting a nephrologist. Appropriate supportive measures and potentially reducing the dose of cisplatin/carboplatin could be an alternative to completely withholding cisplatin, at least in patients without other risk cofactors.

As further potentially modifiable independent factors that limited the administration of chemotherapy, we can also mention organizational, logistical, and administrative reasons, as well as nausea and diarrhea, which also occurred in a relatively significant number of patients. However, without the knowledge of the specific situation and context, it is difficult to draw a clear conclusion, beyond an appeal for prevention, utilization of supportive treatment and care methods, and oversight of the organization and course of procedures for patients undergoing CHRT.

As stated by Multinational Association of Supportive Care in Cancer, optimal supportive care can assist in accurate diagnosis and management, and ultimately improve outcomes; supportive care makes excellent cancer care possible [19].

Regarding limitations, it is necessary to consider the retrospective and single-centre nature of this analysis. However, the robust system of regular daily to weekly check-ups, detailed clinical examinations, comprehensive blood tests, and meticulous electronic documentation ensures a high level of confidence in the recorded data. We also need to take into account that in a small percentage of patients, cisplatin was either replaced with carboplatin (n=6) or fluorouracil (n=1), or its dose was reduced (n=2). These adjustments were made to facilitate the continuation of concomitant chemotherapy when dealing with adverse situations, as outlined in Results. Unfortunately, a certain limitation also lies in the historically non-standard practice of evaluating the differential leukocyte count, and hence, the absolute neutrophil count and neutropenia could not be assessed.

## Conclusions

Aside from the practically unmodifiable poor performance status observed in a small proportion of patients, our analysis primarily identified leukopenia and impaired renal function as the main potentially modifiable independent factors limiting the optimal administration of concomitant CHT in patients with locally advanced cervical cancer undergoing CHRT with IGABT. Other potentially modifiable factors included nausea, diarrhea, anemia, and administrative/organizational issues.

Given the limited guidelines on G-CSF use in CHRT and radiotherapy, further research is certainly needed. In individual cases, we suggest short-acting filgrastim to prevent and manage leukopenia and neutropenia. We also believe that the criteria for 'cisplatin-unfit/ineligible' patients, typically applied in metastatic urothelial carcinoma, should not be automatically adopted for this locally advanced cervical cancer patient cohort due to the significant impact of CHT on treatment outcomes. Therefore, we strongly advocate for maximizing the use of relevant supportive treatment and care methods to ensure the successful completion of concurrent chemotherapy.
